# Epidemiological profile and risk factors associated with death in patients receiving invasive mechanical ventilation in an adult intensive care unit from Brazil: a retrospective study

**DOI:** 10.3389/fmed.2023.1064120

**Published:** 2023-04-25

**Authors:** Camila Vantini Capasso Palamim, Matheus Negri Boschiero, Fernando Augusto Lima Marson

**Affiliations:** ^1^Laboratory of Cell and Molecular Tumor Biology and Bioactive Compounds, São Francisco University, Bragança Paulista, São Paulo, Brazil; ^2^Laboratory of Human and Medical Genetics, Bragança Paulista, São Francisco University, São Paulo, Brazil

**Keywords:** epidemiological profile, intensive care unit, mechanical ventilation, positive end-expiratory pressure, SARS-CoV-2

## Abstract

**Introduction:**

Understanding the epidemiological profile and risk factors associated with invasive mechanical ventilation (IMV) is essential to manage the patients better and to improve health services. Therefore, our objective was to describe the epidemiological profile of adult patients in intensive care that required IMV in-hospital treatment. Also, to evaluate the risks associated with death and the influence of positive end-expiratory pressure (PEEP) and arterial oxygen pressure (PaO_2_) at admission in the clinical outcome.

**Methods:**

We conducted an epidemiological study analyzing medical records of inpatients who received IMV from January 2016 to December 2019 prior to the Coronavirus Disease (COVID)-19 pandemic in Brazil. We considered the following characteristics in the statistical analysis: demographic data, diagnostic hypothesis, hospitalization data, and PEEP and PaO_2_ during IMV. We associated the patients’ features with the risk of death using a multivariate binary logistic regression analysis. We adopted an alpha error of 0.05.

**Results:**

We analyzed 1,443 medical records; out of those, 570 (39.5%) recorded the patients’ deaths. The binary logistic regression was significant in predicting the patients’ risk of death [*X*^2^_(9)_ = 288.335; *p* < 0.001]. Among predictors, the most significant in relation to death risk were: age [elderly ≥65 years old; OR = 2.226 (95%CI = 1.728–2.867)]; male sex (OR = 0.754; 95%CI = 0.593–0.959); sepsis diagnosis (OR = 1.961; 95%CI = 1.481–2.595); need for elective surgery (OR = 0.469; 95%CI = 0.362–0.608); the presence of cerebrovascular accident (OR = 2.304; 95%CI = 1.502–3.534); time of hospital care (OR = 0.946; 95%CI = 0.935–0.956); hypoxemia at admission (OR = 1.635; 95%CI = 1.024–2.611), and PEEP >8 cmH_2_O at admission (OR = 2.153; 95%CI = 1.426–3.250).

**Conclusion:**

The death rate of the studied intensive care unit was equivalent to that of other similar units. Regarding risk predictors, several demographic and clinical characteristics were associated with enhanced mortality in intensive care unit patients under mechanical ventilation, such as diabetes mellitus, systemic arterial hypertension, and older age. The PEEP >8 cmH_2_O at admission was also associated with increased mortality since this value is a marker of initially severe hypoxia.

## 1. Introduction

The intensive care unit (ICU) provides advanced life support to critical patients presenting different severity levels (1). It is, therefore, a specialized facility to monitor and stabilize the patients’ clinical aspects (2). In such a context, critical patients admitted to an ICU might require the use of invasive mechanical ventilation (IMV) to maintain patent airways, improve oxygenation, and prevent aspiration (3, 4). IMV is a complex resource, and the team’s expertise in managing it might generate better results. However, around 38% of the patients that require IMV still die (5). For this reason, knowing the factors that lead to the outcomes of patients under IMV in the ICU is vital to inform the professionals’ conduct better and advise their families (6). Understanding the profile of patients under IMV might lead to decisions such as getting access to technologies, training human resources, and reevaluating care processes, which could allow the structural adjustment of the unit according to the demographic and morbidity characteristics of the population-assisted (7).

Since the appearance of the ICU in 1952, due to the devasting polio epidemic in Copenhagen, the mortality of patients that required care in such units has decreased (8, 9). However, we can consider some factors as death risks, such as male sex, age (elderly), presence of comorbidities (e.g., systemic arterial hypertension, diabetes mellitus, and obesity), and admission diagnosis (e.g., traumatic brain lesion, sepsis, and neurological disorders). Also, it is essential to evaluate the ventilatory parameters at admission, including the positive end-expiratory pressure (PEEP) value, which influences the dissolved oxygen partial pressure in arterial blood (PaO_2_) (10–15).

Regarding ventilatory parameters at admission, the health professionals employed different strategies. However, the literature recommends using protective parameters (low current volumes along with driving pressure and mechanical power limitation) (3, 16, 17). The health professionals used the PEEP to improve oxygenation and stabilize alveolar units when considering ventilatory parameters. Besides that, the PEEP ideal value is still controversial in the scientific literature (18, 19). However, some reports suggest that PEEP ideal values might prevent pulmonary lesions due to the cyclic opening and closing of alveoli. Also, higher values can cause lesions due to alveolar hyperdistention (20).

Some studies considered using 8 cmH_2_O initial PEEP as prophylactic PEEP as a preventive and compensatory value of the functional residual capacity resulting from orotracheal intubation (21). However, when health professionals applied this value to normal lungs, there was no description of improvement in the outcome or time of hospital stay in the scientific literature yet (20, 22). Therefore, according to individual ventilatory mechanics, we must make the best PEEP choice (23). At the same time, PaO_2_ characterizes the degree of hypoxemia and hyperoxemia (24). Both might influence the clinical outcome and length of hospital stay since hypoxemia reduces oxygen supply to tissues. Its cause might have different origins: unbalance in the ventilation/perfusion rate, pulmonary shunt, and hypoventilation. Hyperoxemia, in turn, might cause non-cardiogenic pulmonary edema, formation of hyaline membrane, neutrophilic infiltration, type I pneumocyte damage, type II pneumocyte hyperplasia, alveolar hemorrhage, and an increase in the alveolar sept thickness (25, 26).

This study aimed to describe the epidemiological profile of adult patients admitted to the ICU and receiving IMV at a University Hospital and evaluate the characteristics of the population investigated as risk factors for death and the influence of PEEP and PaO_2_ at admission on the clinical outcome.

## 2. Methods

We carried out an epidemiological study of electronic medical records described in the Philips Tasy® system (Philips Healthcare™), Barueri, São Paulo, Brazil, which records the diagnosis, laboratory data, monitoring of ventilatory support, and clinical evolution of inpatients who required IMV. The patients were included from January 2016 to December 2019. They were assisted at the University Hospital São Francisco de Assis na Providência de Deus ICU, located in Bragança Paulista, São Paulo, Brazil. The ICU has 20 beds for treating critical patients from 15 years old (yo) onwards. The time-period was selected to avoid the Coronavirus Disease (COVID)-19 impact on our data because our University Hospital was a referral center to treat severe cases of Severe Acute Respiratory Syndrome Coronavirus 2 (SARS-CoV-2) infection.

The patients’ characteristics considered in our epidemiological study were: (i) age [years and grouped as adult (18–64 yo) or elderly (>65 yo)], (ii) sex (male and female), (iii) body mass index (BMI) [Kg/m^2^; underweight (<18.5 Kg/m^2^), normal weight (18.5–24.9 Kg/m^2^), overweight (25–29.9 Kg/m^2^), grade I obesity (30–34.9 Kg/m^2^), grade II obesity (35–39.9 Kg/m^2^), and grade III obesity (>40 Kg/m^2^)], (iv) diagnostic (traumatic brain injury, polytraumas, sepsis, elective surgery, acute myocardial infarction, stroke, dyslipidemia, subarachnoid hemorrhage, neuromuscular disease, smoking habits, and others); (v) patient origin from clinics or surgery; (vi) previous history of comorbidities (smoking, alcoholism, cardiopathy, pneumopathy, neurologic sequelae, use of drugs, systemic arterial hypertension, diabetes mellitus, dyslipidemia, and others); (vii) PEEP values at admission in the ICU and during IMV (absolute value and the categorization using the 8 cmH_2_O points as parameter); (viii) PaO_2_ values at admission in the ICU and during IMV [absolute value and the categorization using the following distribution: hypoxia (<80 mmHg), normal (between 80 and 100 mmHg), and hyperoxia (>100 mmHg)]; (ix) length of hospital stay; (x) length of IMV; (xi) presence of ventilation-associated pneumonia; (xii) presence of tracheostomy during hospital stay; and (xiii) outcome (discharge–clinical recovery and death).

Importantly, the protocol used in the admission of patients under mechanical ventilation in the ICU of this study indicates the use of PEEP at levels described in the literature as safe (from 5 to 8 cmH_2_O). Given the need to use higher values, PEEP is titrated according to respiratory mechanics, hemodynamics, and oxygenation indexes. The main purpose of PEEP titration is to maintain alveolar stability and oxygenation at normal levels, that is, to maintain PaO_2_ between 80 and 100 mmHg and peripheral capillary oxygen saturation between 92 and 96%. The tidal volume adopted at the admission of patients was 6 mL/Kg of predicted weight, following the literature recommendations (27).

We performed the descriptive analysis using two approaches. (i) categorical markers–N (%): sample size (percentage); and (ii) numeric markers – mean (standard deviation) and a 95% confidence interval (95%CI) of the mean or median, according to the data distribution, parametric or non-parametric, respectively. We evaluated the normality of the numeric data employing the following three methods: (i) analysis of descriptive measures for central tendency; (ii) plot methods (normal Q-Q plot, trendless Q-Q plot, and boxplot); and (iii) statistical tests (normality tests): Kolmorov-Smirnov test and Shapiro–Wilk test.

The presence of death (categorical data) was associated with the values of the markers with numerical distribution by using the T-test or the Mann–Whitney test. Concomitantly, we associated the death to features with categorical distribution using Fisher’s Exact test or Qui-square test; also, we calculated the relative risk (RR) and the 95%CI for the categorical data. We evaluated Pearson’s correlation coefficient between PaO_2_ and PEEP levels to denote the mutual response. In the Spearman correlations, we considered the following cut-off points: (i) ±0.90–1.00, very strong positive–negative correlation index; (ii) ±0.70–0.89, strong positive–negative correlation index; (iii) ±0.40–0.69, moderate positive–negative correlation index; (iv) ±0.10–0.39, weak positive–negative correlation index; and (v) 0.00–0.09, insignificant (negligible) positive–negative correlation index.

We did the survival curve of patients who received IMV according to PEEP at admission and the classification of PaO_2_ as normal, hypoxia, and hyperoxia at admission. We performed the statistical analysis using the Log-Rank (Mantel-Cose) test. We calculated the Hazard ratio using the PEEP ≤8 cmH_2_O as the numerator.

The binary logistic regression by the stepwise forward method (likelihood ratio) included the patients’ characteristics that presented *p* ≤ 0.05 in the bivariate analysis. However, we excluded the patients’ features with the multicollinearity effect. Also, we excluded BMI and the time when ventilation-associated pneumonia was diagnosed due to a high number of missing data. We considered death a dependent variable, whereas we allocated the other patients’ characteristics as predictors of the risk of death.

We used an alpha error of 0.05, and we did not apply techniques to stipulate the missing data values. We carried out the statistical analysis using the Statistical Package for the Social Sciences version 24.0 (IBM Corp. Released 2015. IBM SPSS Statistics for Windows, version 24.0. Armonk, NY: IBM Corp) software and in the MedCalc software version 15.0 (MedCalc for Windows, version 15.0; MedCalc Software, Ostend, Belgium). Concomitantly, we used the GraphPad Prism software version 8.0 (San Diego, California, United States of America) for figures.

The Ethics Committee of São Francisco University approved the research [CAAE no 29718820.9.0000.5514]. We obtained the waiver of the Informed Consent Term since only the data from the patient’s medical records were obtained without the individual description of the patient.

## 3. Results

### 3.1. Epidemiological profile of patients receiving IMV

We evaluated 3,213 medical records from patients admitted to the ICU. We excluded 1,681 patients since they did not require IMV and 68 since the clinical data was missing. In the initial analysis, we included 1,464 patients who had received IMV. However, we excluded 21 patients later due to the transfer to a different ICU. Thus, we included 1,442 patients in our statistical analysis (Supplementary Figure S1).

We observed a higher frequency of male patients (*n* = 901; 62.4%), adults (*n* = 914; 63.3%), with normal BMI (*n* = 423; 29.3%), or overweight (*n* = 372; 25.8%; Table 1). Among the previous history of comorbidities, the most prevalent were systemic arterial hypertension (*n* = 653; 45.3%), smoking (*n* = 388; 26.9%), diabetes mellitus (*n* = 325; 22.5%), cardiopathy (*n* = 310; 21.5%), neurologic sequel (*n* = 171; 11.9%), alcoholism (*n* = 221; 15.3%), and pneumopathy (*n* = 131; 9.1%; Table 1; Supplementary Table S1).

**Table 1:** Characteristics of the patients in the intensive care unit on invasive mechanical ventilation support during the study period (2016–2019).

Patients’ characteristics	Patients–*N*/1,443 (%)
Age (years)	56.71 ± 17.55; 59 (46–79)
*Age group*	
Adult (18 to 64 yo)	914 (63.3)
Elderly (>65 yo)	529 (36.7)
*Sex*	
Female	542 (37.6)
Male	901 (62.4)
Body Mass Index (Kg/m^2^)	25.92 ± 5.36; 25.60 (22.6–28.8)
Underweight	55 (3.8)
Normal weight	423 (29.3)
Overweight	372 (25.8)
grade I Obesity	139 (9.6)
grade II Obesity	27 (1.9)
grade III Obesity	19 (1.3)
Not informed	408 (28.3)
*Origin*	
Surgery	923 (64.0)
Clinic	520 (36.0)
*Previous history of comorbidities*	
Systemic arterial hypertension	653 (45.3)
Smoking	388 (26.9)
Diabetes mellitus	325 (22.5)
Cardiopathy	310 (21.5)
Alcoholism	221 (15.3)
Neurological sequel	171 (11.9)
Pneumopathy	131 (9.1)
Dyslipidemia	108 (7.5)
Neoplasia	70 (4.9)
Thyroidopathy	70 (4.9)
Kidney disorder	60 (4.2)
Immunodepression	25 (1.7)
Hepatopathy	18 (1.2)
Gastrointestinal disorder	16 (1.1)
Other drugs	49 (3.4)
Other personal backgrounds^*^	45 (3.1)
*Diagnostic*	
Elective surgery	616 (42.7)
Sepsis	375 (26.0)
Cardiopathy	222 (15.4)
Polytrauma	210 (14.6)
Traumatic brain injury	197 (13.7)
Stroke	121 (8.4)
Subarachnoid hemorrhage	104 (7.2)
Acute myocardial infarction	89 (6.2)
Neurologic and psychiatry disorders	69 (4.8)
Nephropathy	31 (2.1)
Neoplasia	23 (1.6)
Other^**^	49 (3.4)
Days of hypoxia	2.57 ± 2.09; 2 (1–3)
Normal days	2.74 ± 2.0; 2 (1–4)
Days of hyperoxia	5.23 ± 4.32; 4 (2–8)
Ventilation-associated pneumonia	410 (28.4)
Tracheostomy	332 (23.0)
Deaths	570 (39.5)

We presented the data as the number of individuals (percentage) and using mean ± standard deviation; median (95% confidence interval). ^*^Supplementary Table S1; ^**^Supplementary Table S2.

A total of 923 (64%) patients were referred to the ICU by the surgery department and the main reason for the admissions were the need for elective surgery (*n* = 616; 42.7%), sepsis (*n* = 375; 26%), cardiopathy (*n* = 222; 15.4%), polytrauma (*n* = 210; 14.6%), and traumatic brain injury (*n* = 197; 13.7%; Table 1; Supplementary Table S2). Ventilation-associated pneumonia occurred in 410 (28.4%) patients, and the need for tracheostomy in 332 (23%) patients; the death of 570 (39.5%) patients was recorded.

### 3.2. Risk factors associated with death in patients receiving IMV

**Figure 1:**
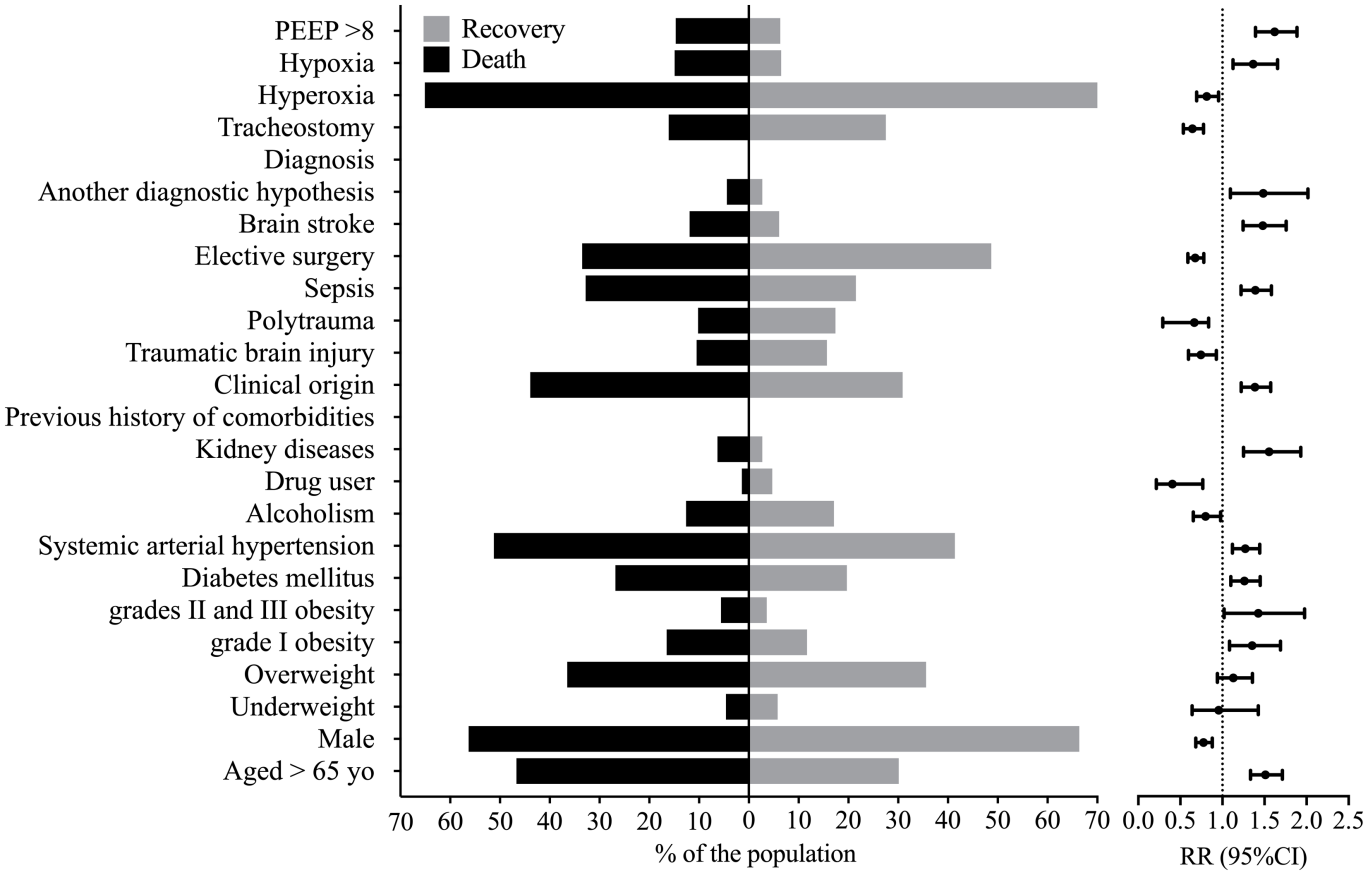
Markers that presented statistical significance in the association between patients that died and those that were discharged from the hospital. This figure shows the percentage of individuals that presented a marker according to the outcome and the relative risk, whose reference was the percentage of individuals discharged from the hospital against the group of patients that died. PEEP, positive end-expiratory pressure; RR, relative risk; yo, years old; 95%CI, 95% confidence interval. We carried out the statistical analysis using the Fisher Exact test or the Chi-square test and a 0.05 alpha error.

Several patients’ characteristics were associated with enhanced lethality, such as older age [RR = 1.512 (95%CI = 1.334–1.713)], enhanced BMI, grades II and III obesity [RR = 1.426 (95%CI = 1.029–1.977)] and grade I obesity [RR = 1.354 (95%CI = 1.085–1.357)], which presented a higher risk of death (Figure 1). Individuals with a previous history of comorbidities of kidney disease [RR = 1.554 (95%CI = 1.251–1.931)], systemic arterial hypertension [RR = 1.271 (95%CI = 1.119–1.443)], and diabetes mellitus [RR = 1.262 (95%CI = 1.099–1.449)] were also at higher risk of death (Supplementary Table S3; Figure 1). The male sex was associated with decreased risk of death when compared to the female sex [RR = 0.776 (95%CI = 0.683–0.880)] (Supplementary Table S3; Figure 1).

**Figure 2:**
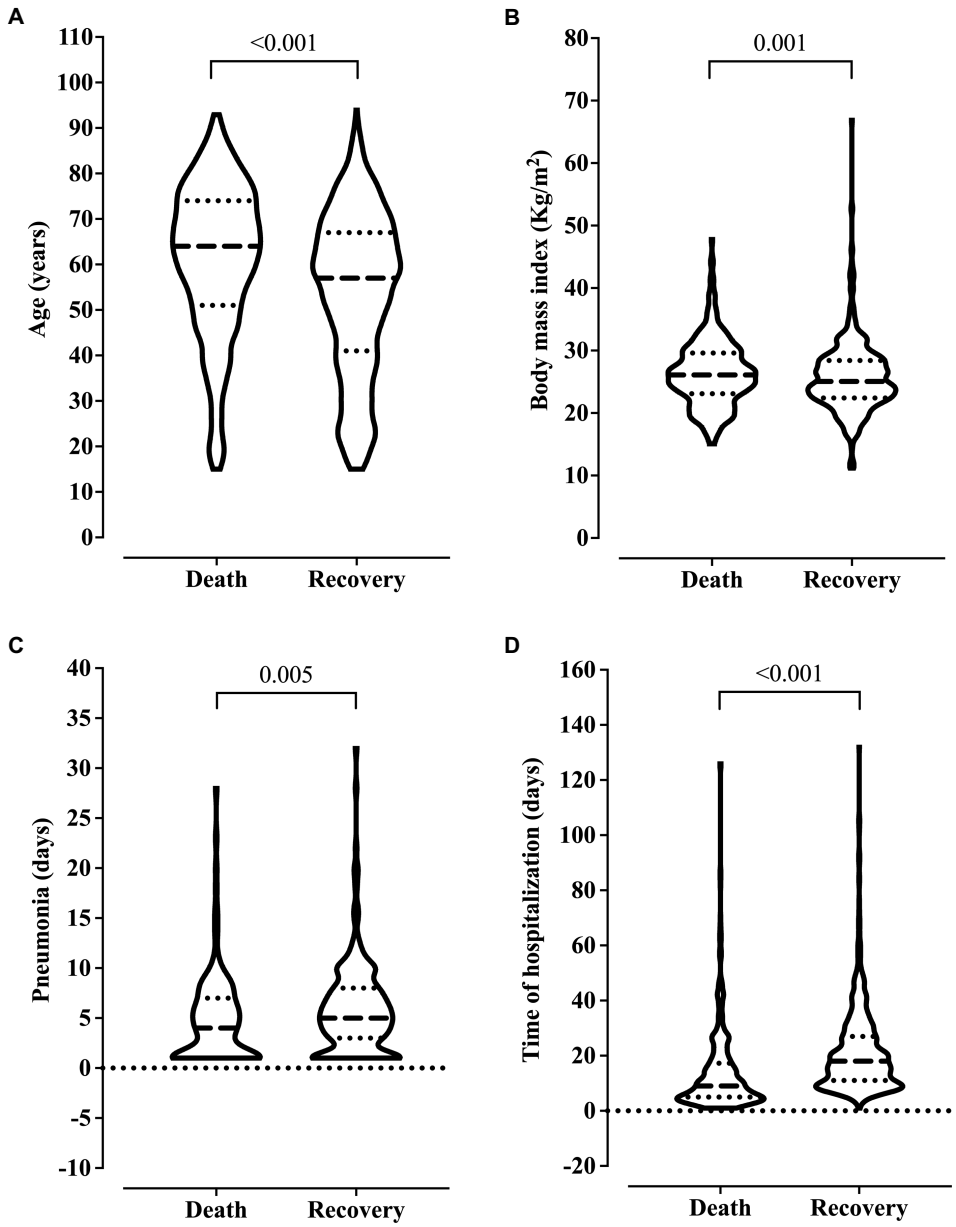
Association between clinical outcome and age **(A)**, body mass index **(B)**, ventilation-associated pneumonia **(C)**, and length of hospital stay **(D)**. We carried out the statistical analysis using the Mann–Whitney test and a 0.05 alpha error.

**Figure 3:**
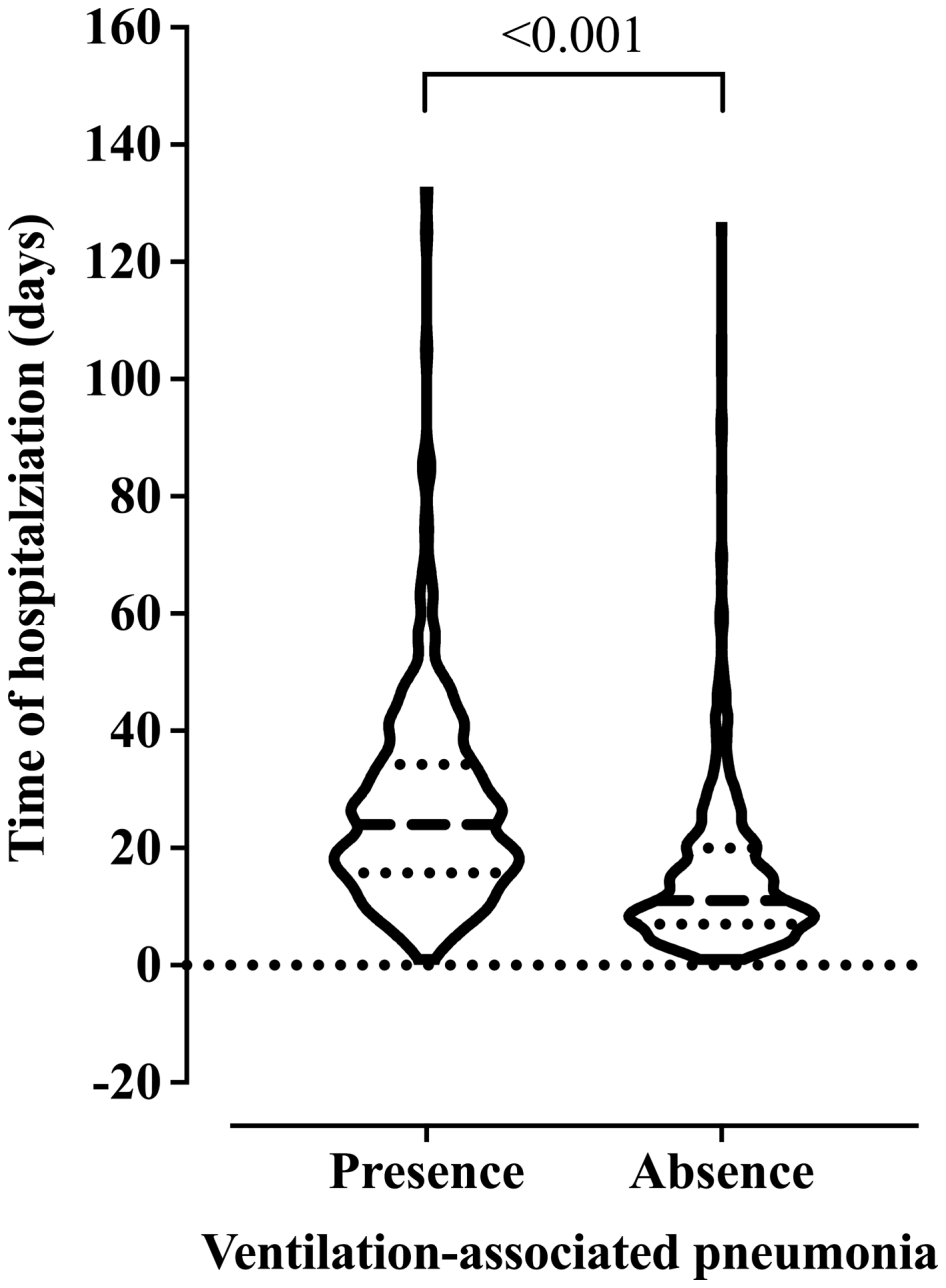
Association between the risk of developing ventilation-associated pneumonia according to the length of invasive mechanical ventilation. We carried out the statistical analysis using the Mann–Whitney test and a 0.05 alpha error.

We observed older age and higher BMI in the patients who died. Also, these patients were hospitalized for more days and diagnosed with ventilation-associated pneumonia earlier than patients who did not die (Figure 2). On the other hand, we related the lowest risk of death to the use of drugs and alcoholism, and the younger age of the patients might explain this finding in this group (data not shown). The presence of pneumonia caused by mechanical ventilation was associated with more extended hospital stays (Figure 3).

Several diagnoses were associated with enhanced lethality such as those from kidney disease [RR = 1.485 (95%CI = 1.094–2.017)], stroke [RR = 1.480 (95%CI = 1.246–1.757)], sepsis [RR = 1.391 (95%CI = 1.222–1.583)], and the clinical origin for the patients [RR = 1.387 (95%CI = 1.223–1.573)] (Supplementary Table S4; Figure 1). However, patients with traumatic brain injury [RR = 0.744 (95%CI = 0.596–0.928)], polytrauma [RR = 0.665 (95%CI = 0.290–0.836)], or those who needed elective surgery [RR = 0.677 (95%CI = 0.589–0.778)] and those who needed tracheostomy [RR = 0.644 (95%CI = 0.535–0.776)] presented a decreased risk of death (Supplementary Table S4; Figure 1); nevertheless, patients who suffered a traumatic brain injury or polytrauma were also younger (data not shown).

### 3.3. Risk of death associated with PEEP and PaO_2_

We associated the PEEP >8 cmH_2_O at admission with a higher risk of death [RR = 1.621 (95%CI = 1.393–1.887)]. In addition, a higher risk of death also occurred in patients with hypoxemia at admission [RR = 1.365 (95%CI = 1.126–1.655)]. In contrast, a lower risk of death occurred in those with hyperoxia [RR = 0.813 (95%CI = 0.693–0.954)] at admission (Supplementary Table S4; Figure 1).

**Figure 4:**
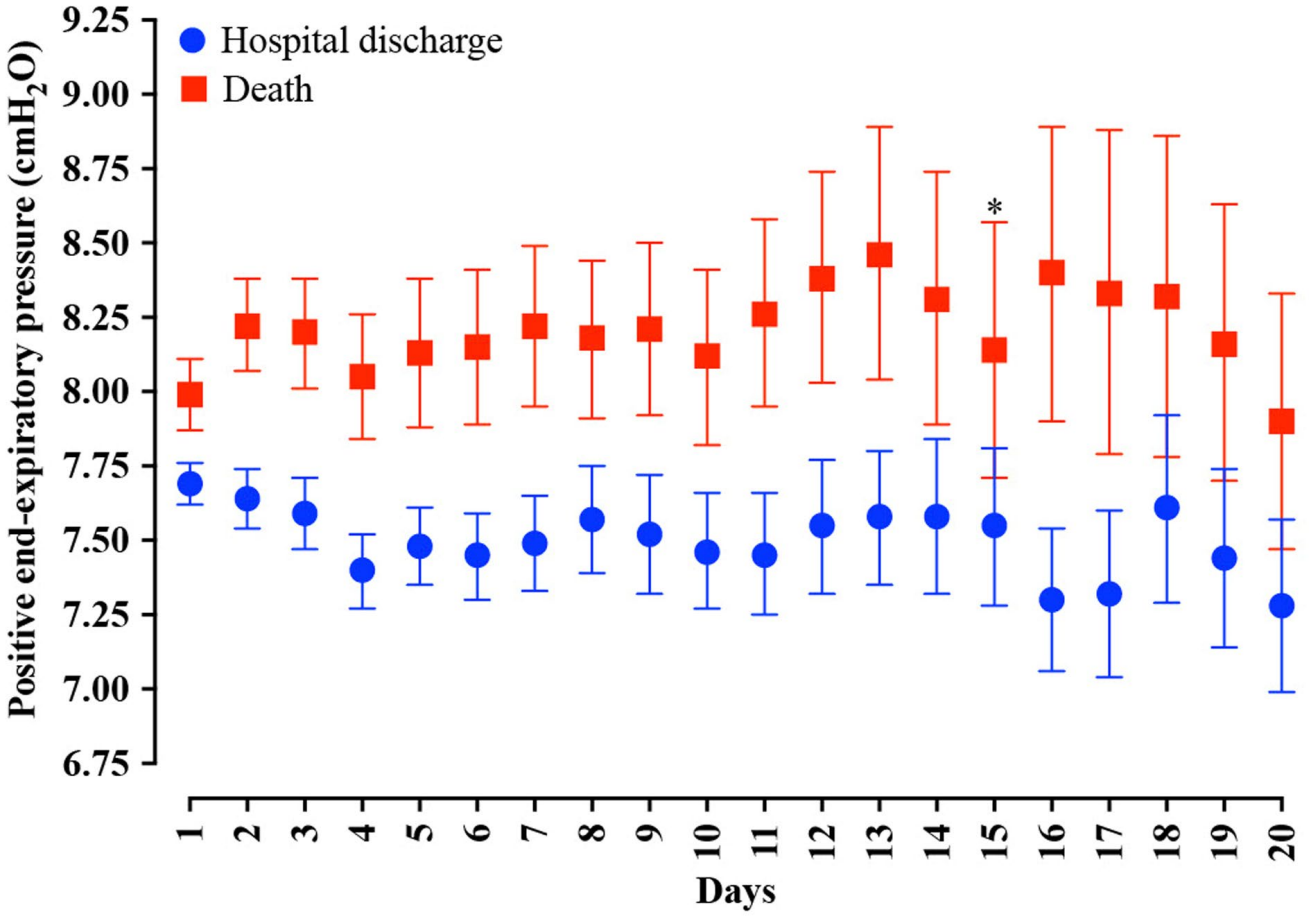
Distribution of the positive end-expiratory pressure (PEEP) values according to the days of invasive mechanical ventilation. Blue represents the individuals that were discharged. Red represents the individuals that died. We carried out the statistical analysis using the Mann–Whitney test and a 0.05 alpha error. The data is presented as mean [95% confidence interval, 95%CI]. *p > 0.05.

**Figure 5:**
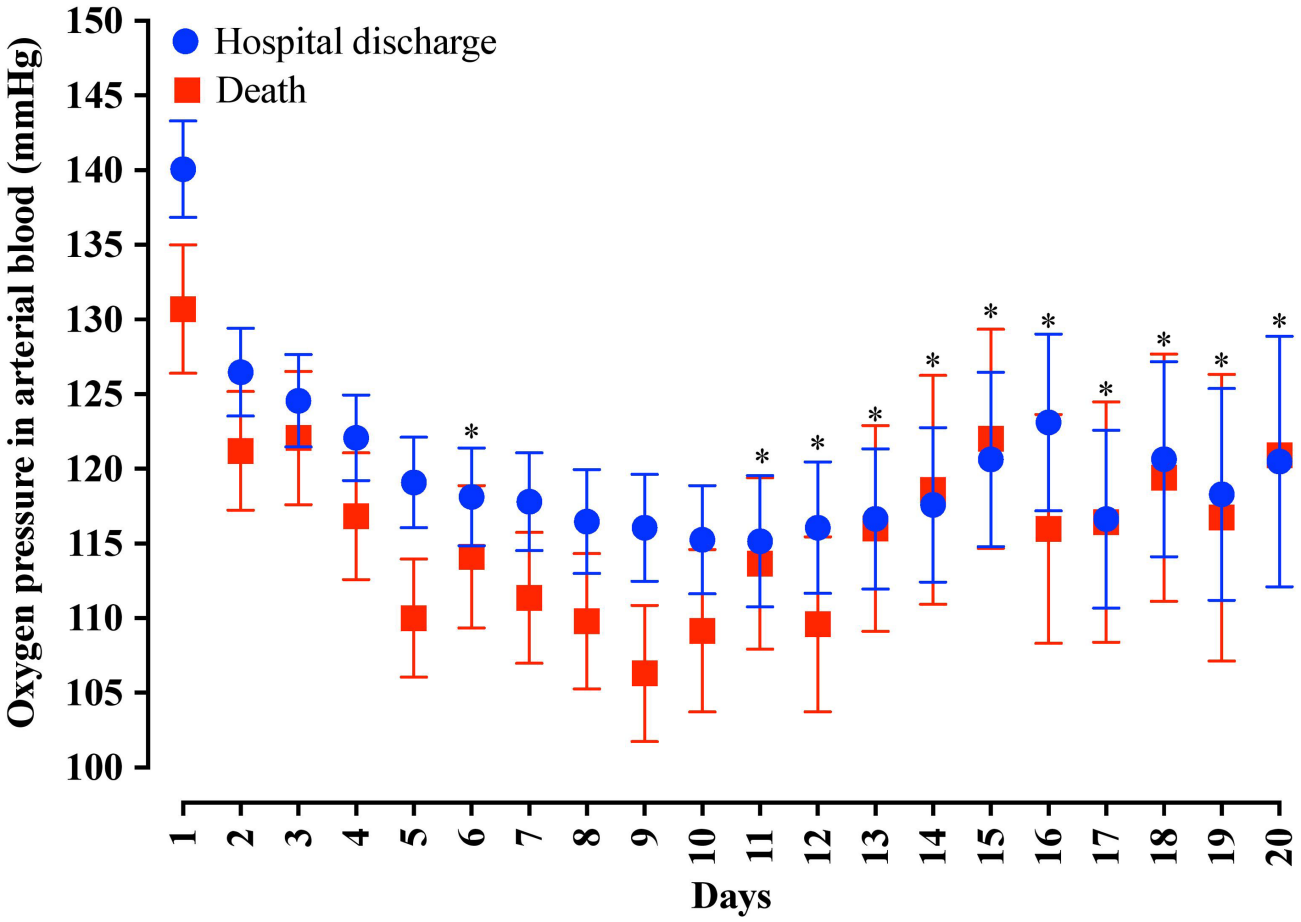
Distribution of the arterial oxygen pressure (PaO_2_) values according to the days of invasive mechanical ventilation. Blue represents the individuals that were discharged. Red represents the individuals that died. We carried out the statistical analysis using the Mann–Whitney test and a 0.05 alpha error. The data is presented as mean [95% confidence interval, 95%CI]. *p > 0.05.

**Figure 6:**
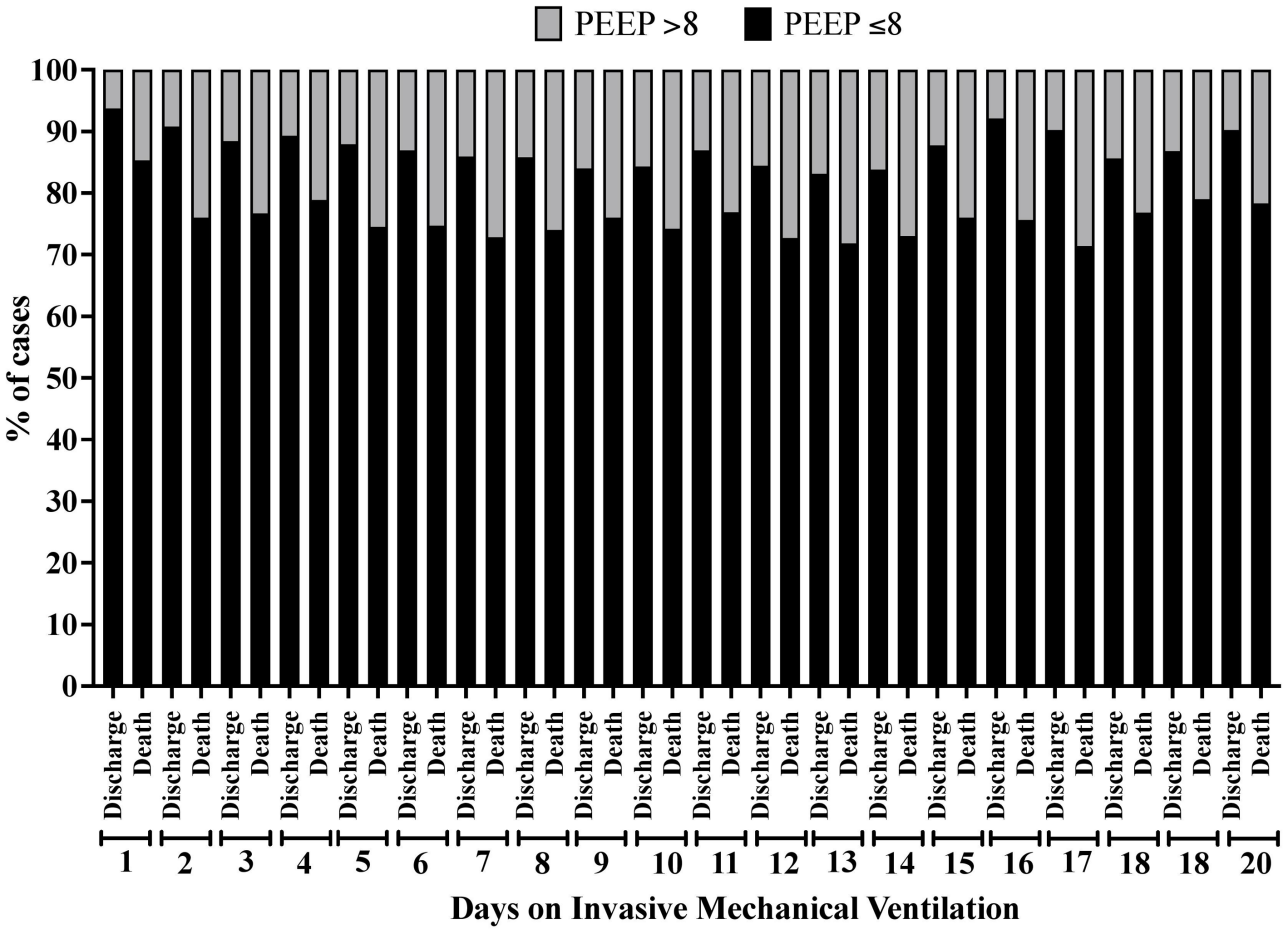
Percentage of patients according to the clinical outcome distributed by the positive end-expiratory pressure (PEEP) value (≤8 cmH_2_O or > 8 cmH_2_O) and according to the time of invasive mechanical ventilation (days 1 to 20).

In the analysis of the first 20 days of intubation, the patients who died required more extended ventilatory support and presented higher PEEP values throughout the first 20 days than those who were discharged, except on the 15^th^ day of hospitalization (Figure 4). Curiously, the PaO_2_ presented lower values in the patients who died between the day of intubation and the 5^th^ day of follow-up and between the 7^th^ and 10^th^ day of intubation (Figure 5). We presented the patients according to the PEEP and the outcome for the 20 days of intubation in Figure 6. It seems relevant to point out that patients who died had more time on PEEP >8 cmH_2_O.

In the Pearson correlation between numeric markers (PEEP at admission, PaO_2_ at admission, IMV duration, length of hospital stay, time until the pneumonia diagnosis, BMI, and age), no statistically significant correlation was observed, except for the correlation between the IMV duration and length of hospital stay (CC = 0.70; *p* < 0.001–strong correlation index), as well as the time until the ventilation-associated pneumonia diagnosis (CC = 0.41; *p* < 0.001–moderate correlation index) and hospital stay (CC = 0.35; *p* < 0.001–weak correlation index) (Supplementary Figure S2).

### 3.4. Survival analysis

**Figure 7:**
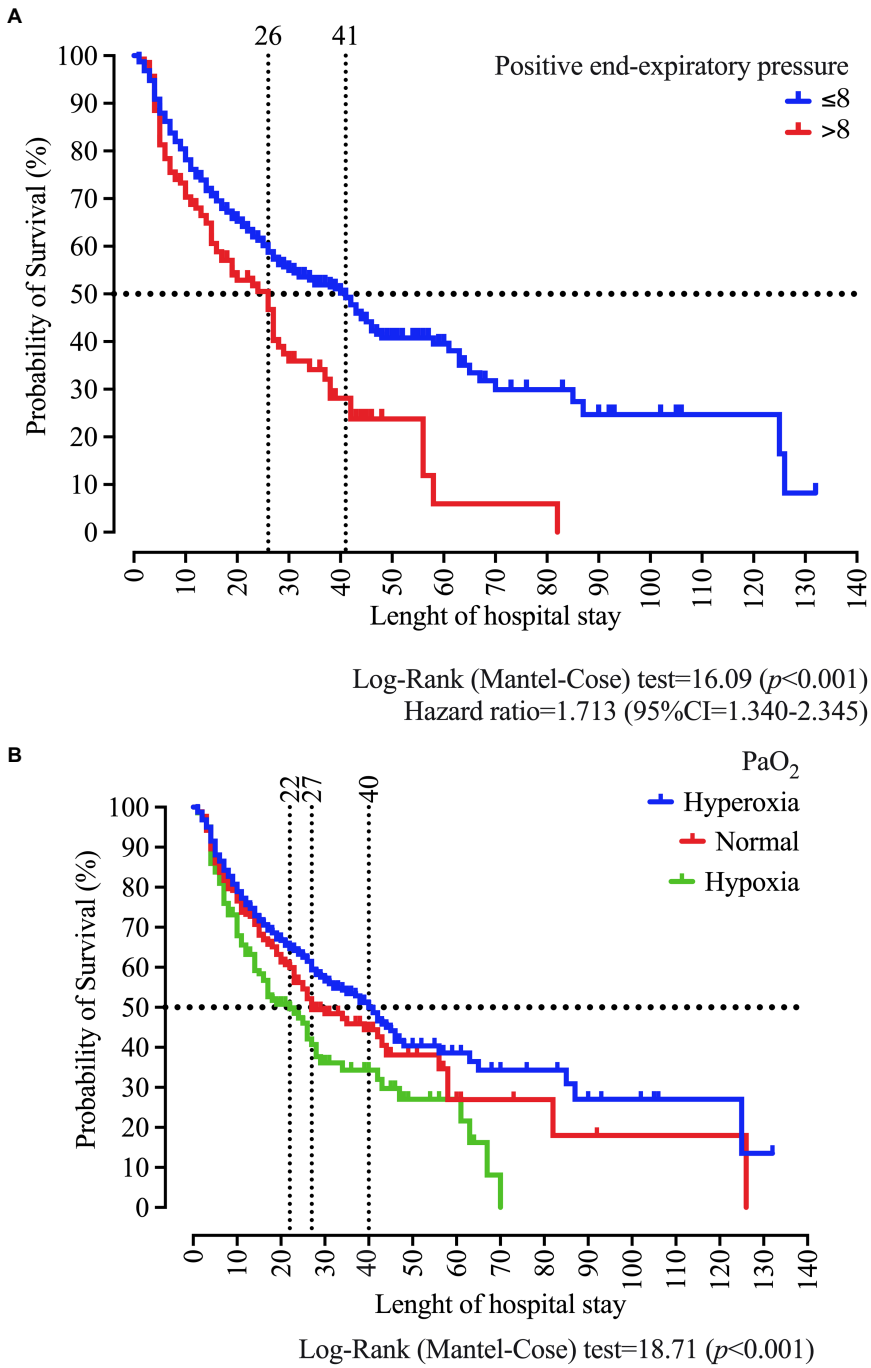
Survival curve of patients who were intubated at the University Hospital according to the positive end-expiratory pressure (PEEP) **(A)**, and the arterial oxygen pressure (PaO_2_) classification as normal, hypoxemia, and hyperoxemia **(B)**. We carried out the statistical analysis using the Log-Rank (Mantel-Cose) test. We calculated the Hazard ratio using the PEEP ≤8 cmH_2_O as the numerator parameter and we adopted a 0.05 alpha error. PaO_2_ values at admission in the ICU were categorized using the following distribution: hypoxia (<80 mmHg), normal (between 80 and 100 mmHg), and hyperoxia (>100 mmHg).

In the survival analysis, we demonstrated that PEEP >8 cmH_2_O at admission is associated with a survival of 26 days. In contrast, we observed in patients with PEEP ≤8 cmH_2_O the survival of 41 days (*p* < 0.001) and a Hazard ratio of 1.713 (95%CI = 1.340–2.345). Regarding the PaO_2_ classification, we found survival values of 40, 27, and 22, respectively, for hyperoxia, normal, and hypoxemia (*p* < 0.001; Figure 7).

### 3.5. Multivariate binary logistic regression analysis

We excluded the BMI and the day of the ventilation-associated pneumonia diagnosis due to a high number of missing data. We also excluded the following markers: previous diagnosis of kidney disease, kidney disease at admission, and use of drugs.

The multivariate analysis by the binary logistic regression performed by the stepwise forward method (likelihood ratio) was significant in determining whether the patients’ characteristics evaluated were likely to predict death [X^2^_(9)_ = 288.335; *p* < 0.001; Nagelkerke *R*^2^ = 0.245]. Predictors that were significant in predicting the risk of death included older age [elderly ≥65 yo; OR = 2.226 (95%CI = 1.728–2.867)]; male sex (OR = 0.754; 95%CI = 0.593–0.959); sepsis (OR = 1.961; 95%CI = 1.481–2.595); need for elective surgery (OR = 0.469; 95%CI = 0.362–0.608); stroke (OR = 2.304; 95%CI = 1.502–3.534); length of hospital stay (OR = 0.946; 95%CI = 0.935–0.956); hypoxemia (OR = 1.635; 95%CI = 1.024–2.611), and PEEP >8 cmH_2_O at admission (OR = 2.153; 95%CI = 1.426–3.250). In contrast, hyperoxia could not predict the risk of death (Table 2).

**Table 2:** Multivariate binary logistic regression analysis predicts the death of adult and old patients admitted to an intensive care treatment unit.

Predictors	B	SE	Wald	df	Sig	Exp(B)	95%CI	
							Lower limit	Upper limit
Age (Elderly)	0.800	0.129	38.329	1	<0.001	2.226	1.728	2.867
Sex (Male)	−0.283	0.123	5.307	1	0.021	0.754	0.593	0.959
Sepsis (Positive)	0.673	0.143	22.136	1	<0.001	1.961	1.481	2.595
Elective surgery (Presence)	−0.757	0.132	32.774	1	<0.001	0.469	0.362	0.608
Cerebrovascular accident (Positive)	0.834	0.218	14.615	1	<0.001	2.304	1.502	3.534
Length of hospital stay (days)	−0.056	0.006	99.131	1	<0.001	0.946	0.935	0.956
PaO_2_ (normal)			14.712	2	0.001			
PaO_2_ (Hyperoxemia)	−0.273	0.157	3.016	1	0.082	0.761	0.560	1.036
PaO_2_ (Hypoxemia)	0.492	0.239	4.245	1	0.039	1.635	1.024	2.611
PEEP (>8 cmH_2_O)	0.767	0.210	13.320	1	<0.001	2.153	1.426	3.250
Constant	0.525	0.200	6.853	1	0.009	1.690		

Variables not inserted in the equation using the stepwise forward method: patient’s origin (surgery or clinic); traumatic brain injury; polytrauma; cerebrovascular accident; the presence of ventilation-associated pneumonia; the need for tracheostomy; diabetes mellitus; systemic arterial hypertension; and alcoholism. B, regression coefficient estimated for the predictor; SE, regression coefficient standard error; df, degrees of freedom; Exp(B), odds ratios for the predictors; CI, confidence interval; PEEP, positive end-expiratory pressure; PaO_2_, arterial oxygen pressure. PaO_2_ values at admission in the ICU were categorized using the following distribution: hypoxia (<80 mmHg), normal (between 80 and 100 mmHg), and hyperoxia (>100 mmHg).

## 4. Discussion

This study described the death of 570 patients (39.5%) during ICU stay on IMV at a University Hospital. A higher risk of this outcome occurred in patients that presented older age, sepsis diagnosis, presence of cerebrovascular accident, hypoxemia at admission, and the use of PEEP >8 cmH_2_O at admission. The epidemiological profile of patients admitted to the adult ICU of the University Hospital shows mainly adult male patients with a previous history of diabetes mellitus, systemic arterial hypertension, alcoholism, and smoking habits. Those patients were usually referred to the ICU by the surgical team, including those undergoing elective surgeries (42.7%). The leading causes of admission to the ICU included traumatic brain injury, polytrauma, and sepsis. During the follow-up period, 410 (28.4%) patients presented ventilation-associated pneumonia.

### 4.1. Epidemiological profile of patients receiving IMV and death risk

This study found a 39.5% death rate, which is only associated with patients receiving IMV. In the literature, a multicenter study that analyzed data from 361 ICUs located in the United States of America, Europe, and Latin America and included 5,183 individuals receiving mechanical ventilation reported a 52% death rate in patients that required mechanical ventilation due to respiratory insufficiency (7). Another two studies performed in Brazil and Chile also reported a high prevalence of death in ICU patients who required mechanical ventilation, with 34 and 33.9%, respectively (28, 29). Interestingly, the overall in-hospital mortality in the Brazilian study was higher than in ICU (42% vs. 34%). Our study presented a similar demographic profile to those found in the literature, which showed the prevalence of male patients, and older individuals to be higher in ICU patients with mechanical ventilation; in contrast, our study showed the main causes of mechanical ventilation to be surgery followed by pneumonia, cardiopathy, sepsis, and trauma. Those authors also reported that the factor that leads to the need for mechanical ventilation might influence the outcome. In Brazil, most patients in ICU are male (50.78%) (30), which is similar to the ones found in the United States of America (51.5%) and the United Kingdom (57.2%) (30, 31). As for the age range, adult individuals prevail in Brazil and the United States of America (30, 31).

In this study, we associated the presence of older age, obesity, systemic arterial hypertension, diabetes mellitus, and kidney insufficiency with a higher likelihood of death. This data follows the literature (7, 28, 29). Curiously, these markers seem to be part of the profile of the patients assisted in Brazil since, according to the Brazilian Intensive Medicine Association, the most frequent comorbidities found in patients admitted to ICU in the country include systemic arterial hypertension (66.40%), diabetes mellitus (32.82%), and kidney disorders (11.63%). The prevalence of male patients was also reported (51.30%) by that institution (30). Such comorbidities might lead to the risk of ICU admission, in which diabetes mellitus, for example, is associated with an increased risk of infection in several sites (skin, nervous system, bones, and articulations) (32). Systemic arterial hypertension, in turn, is the most critical morbidity and mortality risk factor in the world and is associated with an increased risk of cardiovascular diseases (33). Finally, kidney insufficiency presents a 57% increase in the mortality risk of critical patients due to its consequences, namely, metabolic acidosis, electrolytic unbalance, and uremic toxicity (34).

Obesity is also a predictor of extended hospital stay since it might affect several organs, mainly the lungs and heart. In addition, it requires differentiated mechanical ventilation management and higher ventilatory weaning expertise (34). The literature reports a relevant study carried out in the United Kingdom, including over 3.6 million individuals, which pointed out higher death incidence in patients with BMI over the band considered healthy [BMI >30 Kg/m^2^ (obesity)]. However, that study identified the influence of age and BMI and reported that low BMI increases death risk in young individuals. At the same time, a higher BMI might have a protective effect in older people (which might be associated with higher nutritional reserve) (35). However, several meta-analyses and other studies have reported that obesity mainly influences the length of hospital stay rather than death risk (36–40). Maybe the obesity variable is part of a Simpson paradox, that is, in which a certain tendency disappears or even reverses when groups are combined, which is perhaps related to the difficulty in asses the severity of patients with obesity in ICU since the most used scores (APACHE II or III, Sequential Organ Failure Assessment and Simplified Acute Physiology Score II) do not take into account patients weight (41–43). Additionally, APACHE II score in individuals with obesity might be over or underestimate since the patients tend to have a low alveolar gradient, mainly due to a higher prevalence of hypoventilation and apnea syndrome, and also have low urine output, leading to an illusory increased kidney dysfunction (41, 44). The obesity role in the outcome of patients admitted to the ICU, mainly those requiring IMV, still needs further studies since a new “pandemic” of individuals with obesity has been observed worldwide (45). Studies also need to assess the Simpson paradox, which could bias the analysis.

It seems relevant to emphasize that comorbidities do not always develop individually; therefore, when considered together, they might increase the likelihood of adverse outcomes even more. It is essential to highlight that the risk factors can be modifiable and reduced by public health policies, awareness-raising, and better access to health services. Implementing campaigns incentivizing healthy eating habits, regular physical exercises, adherence to disease control measures, and stopping smoking and consuming alcohol help manage those diseases. For example, these actions aim to reduce the incidence of obesity, systemic arterial hypertension, and diabetes mellitus and, consequently, might reduce the occurrence of cardiovascular events (46).

Regarding diagnosis at admission, our study shows that patients in treatment with sepsis, cerebrovascular accident, and kidney disorders also present a higher death risk than individuals diagnosed with traumatic brain injury, polytrauma, elective surgeries, and those that evolved to tracheostomy. Some findings in our study disagree with those in the literature since patients with traumatic brain injury and polytrauma were younger than other patients. For example, the cerebrovascular accident, along with the need for mechanical ventilation, presents a high mortality rate (56.6%) and tends to predominate among male patients (52.7%) with a mean age of 60 yo (47, 48). We confirmed this data in our study, which showed that male sex, diagnosis of cerebrovascular accident, and age are more frequent among our patients; however, male sex was not a death predictor in our data.

When considering death risk markers, sepsis is responsible for ~30–60% of deaths in the ICU (49). The highest death risk due to sepsis results from organ failure caused by the host’s deregulated response to the infection. Despite all efforts made to prevent infections and treat patients affected by them, sepsis is still one of the most common causes of death worldwide, with varied rates according to the region (South Africa and Asia are the most affected regions), age (older age is more associated with death risk), and sex (male) (24, 50, 51). As for treatment, empirical antimicrobial therapy is still the base treatment, and its start is indicated in the first 6 h of the diagnosis. Each hour of delay in the treatment represents a 6% increase in the death risk. The literature described that prescribing unsuitable antimicrobial drugs increases death rates and bacteria resistant to antibiotic medication. In addition, antibiotic medicine might eliminate the bacteria from the blood plasma. However, it might not be efficient in preventing pathogen proliferation in the erythrocyte, which might cause the inefficiency of some treatments against sepsis (52). The sepsis profile described is similar to the profile observed in patients assisted at the University Hospital where we carried out the study.

Elective surgeries that require ICU admission represent 9.7% of this treatment. Of those, ~50.4% also present postoperative complications [e.g., pulmonary embolism and cardiac arrest], with a mortality rate from 2.4 to 9.7% (53). We can associate the lower death risk after elective surgery with the preparation that precedes the procedure.

### 4.2. Death risk associated with PEEP and PaO_2_

This study described the highest death risk of patients receiving ventilation with PEEP >8 cmH_2_O, maintaining hypoxemia. On the other hand, patients with hyperoxemia showed a lower death risk. Some studies have pointed out that PEEP does not reduce the incidence of pulmonary complications and should not be considered a protective factor for a favorable outcome. In addition, PEEP might increase oxygenation; however, in other cases, it might lead to static stretching, resulting in lesions (18, 54). A study carried out the analysis of surgical patients. It showed that PEEP use resulted in a 5% death risk reduction due to decreased postoperative pulmonary complications such as atelectasis and hypoxemia. However, those findings were inconclusive due to research limitations (small sample) (55). Concomitantly, we observed a higher survival rate in patients that used PEEP ≤8 cmH_2_O. However, the outcome does not seem to be associated with the PEEP cut-off point in the literature (22, 23). Gatinoni and co-workers (2015) concluded that there is not “a PEEP correct value” and that it must be titrated by taking into consideration several factors (e.g., oxygenation and hemodynamics) (19). In addition, PEEP is not the only risk factor associated with worse outcomes among the ventilator parameters. In this context, it is important to evaluate parameters such as tidal volume, driving pressure, and plateau pressure that can improve the risk of ventilator-induced lung injury (known as barotrauma and volutrauma), which in turn increase the risk of death (56). Importantly, the two terms–barotrauma and volutrauma–reflect the two sides of the same phenomenon: lung injury due to a large distending volume and/or to a high airway pressure.

In extreme cases, hypoxemia might lead to organ failure (57). In contrast, hyperoxemia might lead to acute hyperoxic and acute lung injury, damaging the epithelium and endothelium due to the release of pro-inflammatory cytokines [e.g., Tumor Necrosis Factor Alpha (TNF-α) and Interferon Gamma (IFN-g)], which might start a pulmonary injury process (25, 58). Although hyperoxemia in the first 24 h of hospital admission does not seem to increase death risk in severe trauma patients (59), it is associated with a higher death risk in patients with cardiorespiratory arrest (60). The literature associated the use of supplementary oxygen in patients with hyperoxemia (PaO_2_ over 150 mmHg) with the worst clinical outcome, possibly due to vasoconstriction, reduction in the coronary blood flow, and cardiac output, the release of free radicals, and microvascular perfusion modulation (58, 61).

Despite the general reduction in death risk in patients with PaO_2_ over 150 mmHg in the first 24 h of ICU admission, high PaO_2_ values should not be recommended when we know the etiology of the tissue oxygenation decrease (e.g., due to hampered transportation). Thus, it might not be wise to state that high levels of arterial oxygenation are always beneficial or might cause harmful side effects (62). In addition, the goals of applying PEEP are to improve gas exchange and increase functional residual capacity, but the effects of PEEP on heart function include reduced venous return, increase pulmonary vascular resistance, and afterload to the right heart, which can lead to worsening oxygenation (63).

### 4.3. Multivariate binary logistic regression analysis

We identified the following markers as the main predictors for death: female sex, elderly, sepsis, cerebrovascular accident, hypoxemia, and PEEP >8 cmH_2_O. Concomitantly, patients undergoing elective surgery and male sex presented lower chances of death.

We developed the study at a trauma referral center in the region. This fact could lead to an increase in the death risk in male patients, which would confirm other epidemiological studies on trauma centers in Brazil (located in Parana, Bahia, and Paraiba states). However, the male sex was associated with the lowest death incidence. A fact that could explain our findings is that these male patients might have had their age as the primary protective factor since they were all younger patients (data not presented).

Among the elderly, traumatic brain injury might increase mortality when associated with several comorbidities, such as falls, which can even contribute to the cause of trauma (64–66). A retrospective cohort study that analyzed data from 8,598 patients reported that most ICU admissions were male patients. However, the analysis did not show a difference between the sexes when comparing the length of hospital stays, but the hospital discharge rate was higher for female patients (67). In addition, older patients are more vulnerable and might develop multiple organ failures faster, leading to an increased death rate in that population (68).

Sepsis is accountable for 25% of ICU admissions in Brazil and shows high mortality rates, which might reach 65%, while sepsis mortality means around the world might reach 40% (69). Being an organ failure caused by the deregulated and unsuitable host response to infection, sepsis is potentially fatal, and its mortality rate is higher in environments of low or medium resources (70).

Elective surgeries usually present a low mortality rate (between 1 and 4%), and pre-operative care procedures are essential for safe surgical treatment. However, the ideal level of such care has not been defined yet, and death still occurs, mainly due to postoperative complications, for example, pulmonary embolism and cardiac arrest (53).

Both hypoxemia and the use of PEEP >8 cmH_2_O were factors that increased mortality rates in our analysis. A study developed with rats that analyzed PEEP to prevent postoperative pulmonary complications reported that PEEP >8 cmH_2_O prevented such complications (71). However, that study reported a postoperative analysis only. In addition, regarding PaO_2_, health professionals are most concerned with hypoxemia than with the harmful effects of hyperoxemia. For this reason, PaO_2_ at admission is oftenly higher than recommended. However, the mortality curve related to PaO_2_ at admission presents a U shape. The mortality risk increases as much with low PaO_2_ as with high. Also, it is relevant to highlight that the oxygen supplementary offer and the PEEP influence the PaO_2_ (72). Although PEEP reduces the collapse of alveolar units and the incidence of atelectasis, one of the factors causing hypoxemia (73), the use of high PEEP values might lead to injury induced by static stretching of alveolar units, mainly when we consider the time in mechanical ventilation support since it is usually longer in patients of clinical or trauma origin (18, 74). The PEEP ideal value remains an unanswered question, and if underestimated, it might collapse the alveoli hampering gas exchange. On the other hand, if overestimated, it might lead to alveolar hyperdistention, inhibiting gas exchange, and venous return (19, 20). Therefore, we must compare PEEP titration to the drug administration, which must be applied rationally based on the patient’s condition.

PEEP increases linearly the mechanical power, which is the energy delivered to the alveolus because of the ventilatory parameters set (75). The mechanical power equation might help the clinical team to estimate injuries associated with mechanical ventilation support by observing the variables present in its formula (current volume, respiratory rate, and inspiratory time). Since PEEP increases the mechanical power volume linearly, it also increases the risk of injury associated with ventilation and death (56). Our study showed increased death risk with PEEP >8 cmH_2_O, which might be related to lesions caused by the ventilation, which agrees with the literature.

A recent study incorporated PEEP into the PaO_2_/FiO_2_ ratio to evaluate the mortality predisposition of patients receiving mechanical ventilation, and it was seen to be a good marker. That study also reported that PEEP incorporated into the PaO_2_/FiO_2_ ratio alters the classification of gas exchange severity in critical patients (76). The pandemic caused by the new coronavirus (COVID-19) raised great interest in PEEP since this disease affects the lungs severely in some cases leading to a condition like that of acute respiratory discomfort syndrome, requiring better mechanical ventilation performance (77).

## 5. Limitations

The limitations of our study include a small sample and missing data such as the absence of severity score (e.g., APACHE II or III, Sequential Organ Failure Assessment and Simplified Acute Physiology Score II) and some values for BMI, and pneumonia associated with ventilation. Data such as tidal volume, driving pressure, and oxygenation index were not collected because the objective of this study was to evaluate the PEEP influence and PaO_2_ separately from the other parameters. We performed an observational study, which might lead to confounding factors. In addition, due to the COVID-19 pandemic, the 2020 and 2021 data were not included since the pandemic modulated and affected ICU admissions, including referred ICU (78–81). Finally, our study is a picture coming from the Brazilian scenario and this could or could not match exactly the worldwide scenario, including access to the health system and/or the standard of admissible patients to treatment in ICU, together with code-status regulations. Also, in the future, it is important to perform other observational studies as those performed by National Institute for Health and Care Research Global Health Unit on Global Surgery and COVIDSurg Collaborative to improve the world’s capacity to deal with conditions such as COVID-19 pandemic and its impact on the health system, including ICUs collapse during the COVID-19 pandemic and the comparison between time-lapse periods (before-, during-, and after-COVID-19 pandemic) (78–82).

## 6. Conclusion

The death rate of the studied ICU was equivalent to that of other similar units. Regarding risk predictors, several demographic and clinical characteristics were associated with enhanced mortality in ICU patients under mechanical ventilation, such as diabetes mellitus, systemic arterial hypertension, and older age. The PEEP >8 cmH_2_O at admission was also associated with increased mortality since this value is a marker of initially severe hypoxia.

## Data availability statement

The original contributions presented in the study are included in the article/Supplementary material, further inquiries can be directed to the corresponding author.

## Ethics statement

The Ethics Committee of São Francisco University approved the research [CAAE no 29718820.9.0000.5514]. Written informed consent from the [patients/participants OR patients/participants legal guardian/next of kin] was not required to participate in this study in accordance with the national legislation and the institutional requirements.

## Author contributions

All authors listed have made a substantial, direct, and intellectual contribution to the work and approved it for publication.

## Funding

MNB received a grant from FAPESP (Brazilian acronym for *Fundação de Amparo à Pesquisa do Estado de São Paulo* [São Paulo Research Foundation]; no 2021/05810–7).

## Conflict of interest

The authors declare that the research was conducted in the absence of any commercial or financial relationships that could be construed as a potential conflict of interest.

## Publisher’s note

All claims expressed in this article are solely those of the authors and do not necessarily represent those of their affiliated organizations, or those of the publisher, the editors and the reviewers. Any product that may be evaluated in this article, or claim that may be made by its manufacturer, is not guaranteed or endorsed by the publisher.
