# Supplementary material for: Epidemiological profile and risk factors associated with death in patients receiving invasive mechanical ventilation in an adult intensive care unit from Brazil: a retrospective study
Source: Front Med (Lausanne). 2023 Apr 25;10:1064120. doi: 10.3389/fmed.2023.1064120 (PMC10166862; doi:10.3389/fmed.2023.1064120)
Supplement: Supplementary file 1 [file Data_Sheet_1.docx]

**Title:** Epidemiological profile and risk factors associated with death in patients receiving invasive mechanical ventilation in an adult intensive care unit from Brazil: A retrospective study

| **Supplementary Material – Table 1.** Previous history of comorbidities of the patients in the intensive care unit on invasive mechanical ventilation during the study period (2016-2019). | |
| --- | --- |
| **Characteristics** | **Patient – N** |
| Arthrosis | 5 |
| Anemia | 5 |
| Deep vein thrombosis | 3 |
| Glaucoma | 3 |
| Malnutrition | 3 |
| Obstructive sleep apnea | 3 |
| Amputation | 2 |
| Arthrosis and anemia | 1 |
| Deafness | 1 |
| Down syndrome | 1 |
| Ectopic pregnancy | 1 |
| Fibromyalgia | 1 |
| Hearing deficiency | 1 |
| Hernia | 1 |
| Hysterectomy | 1 |
| Leprosy | 1 |
| Malnutrition and anemia | 1 |
| Miscarriage | 1 |
| Prostatectomy | 1 |
| Puerperal | 1 |
| Pulmonary thromboembolism | 1 |
| Sacral scab | 1 |
| Sleep apnea | 1 |
| Sickle cell anemia | 1 |
| Thrombocytosis | 1 |
| Tracheostomy | 1 |
| Transient ischemic attack | 1 |
| Traumatic brain injury | 1 |
| N, number of individuals. |  |

| **Supplementary Material – Table 2.** Diagnosis of the patients in the intensive care unit on invasive mechanical ventilation during the study period. | |
| --- | --- |
| **Diagnosis** | **Patient – N** |
| Chest drainage | 3 |
| Neuromuscular disease | 3 |
| Pulmonary thromboembolism | 3 |
| Urinary infection | 3 |
| Cardiorespiratory arrest | 2 |
| Chronic obstructive pulmonary disease | 2 |
| Motor sequelae | 2 |
| Abscess | 1 |
| Asthma | 1 |
| Colostomy | 1 |
| Diabetes Insipidus | 1 |
| Diverticulitis | 1 |
| Drowning | 1 |
| Granulomatosis with polyangiitis | 1 |
| High digestive bleeding | 1 |
| Human immunodeficiency virus (HIV) infection | 1 |
| Intoxication | 1 |
| Lower gastrointestinal bleeding | 1 |
| Lowered level of consciousness | 1 |
| Miscarriage | 1 |
| Multiple organ disfunction | 1 |
| Pneumocystosis | 1 |
| Pneumothorax | 1 |
| Pulmonary emphysema | 1 |
| Severe acute respiratory infection and Multiple organ disfunction | 1 |
| Systemic inflammatory response syndrome | 1 |
| Thrombophilia | 1 |
| Not informed | 11 |
| N, number of individuals. |  |

| **Table 3.** Association between demographic markers and personal background of patients admitted to the intensive care unit on invasive mechanical ventilation as a death risk factor. | | | | | | | |  |
| --- | --- | --- | --- | --- | --- | --- | --- | --- |
| **Patient’s characteristics** | **Groups** | **Deaths – N (%)** | **Discharge – N (%)** | **Total – N** | ***p*** | **RR** | **95%CI** |  |
| Age group | Adult | 304 (53.3) | 610 (69.9) | 914 | 0.001 | Reference | - |  |
|  | Elderly | 266 (46.7) | 263 (30.1) | 529 |  | 1.512 | 1.334-1.713 |  |
| Sex | Female | 249 (43.7) | 293 (33.6) | 542 | 0.001 | Reference | - |  |
|  | Male | 321 (56.3) | 580 (66.4) | 901 |  | 0.776 | 0.683-0.880 |  |
| Body mass index | Underweight | 18 (4.6) | 37 (5.8) | 55 | 0.046 | 0.955 | 0.639-1.426 |  |
|  | Normal weight | 145 (36.8) | 278 (43.4) | 423 |  | Reference | - |  |
|  | Overweight | 144 (36.5) | 228 (35.6) | 372 |  | 1.129 | 0.940-1.357 |  |
|  | Obesity – grade I | 65 (16.5) | 74 (11.5) | 139 |  | 1.354 | 1.085-1.690 |  |
|  | Obesity – grades II and III | 22 (5.6) | 24 (3.7) | 46 |  | 1.426 | 1.020-1.977 |  |
| Personal background |  |  |  |  |  |  |  |  |
| Diabetes mellitus | Absent | 417 (73.2) | 701 (80.3) | 1.118 | 0.002 | Reference | - |  |
|  | Present | 153 (26.8) | 172 (19.7) | 325 |  | 1.262 | 1.099-1.449 |  |
| Systemic arterial hypertension | Absent | 278 (48.8) | 512 (58.6) | 790 | 0.001 | Reference | - |  |
|  | Present | 292 (51.2) | 361 (41.4) | 653 |  | 1.271 | 1.119-1.443 |  |
| Smoking | Absent | 426 (74.7) | 629 (72.1) | 1,055 | 0.275 | Reference | - |  |
|  | Present | 144 (25.3) | 244 (27.9) | 388 |  | 0.919 | 0.792-1.067 |  |
| Alcoholism | Absent | 498 (87.4) | 724 (82.9) | 1,222 | 0.025 | Reference | - |  |
|  | Present | 72 (12.6) | 149 (17.1) | 221 |  | 0.799 | 0.654-0.978 |  |
| Other drugs | Absent | 562 (98.6) | 832 (95.3) | 1,394 | 0.001 | Reference | - |  |
|  | Present | 8 (1.4) | 41 (4.7) | 49 |  | 0.405 | 0.214-0.766 |  |
| Dyslipidemia | Absent | 529 (92.8) | 806 (92.3) | 1,335 | 0.760 | Reference | - |  |
|  | Present | 41 (7.2) | 67 (7.7) | 108 |  | 0.958 | 0.746-1.230 |  |
| Pneumopathy | Absent | 508 (89.1) | 804 (92.1) | 1,312 | 0.061 | Reference | - |  |
|  | Present | 62 (10.9) | 69 (7.9) | 131 |  | 1.222 | 1.008-1.483 |  |
| Cardiopathy | Absent | 434 (76.1) | 699 (80.1) | 1,133 | 0.077 | Reference | - |  |
|  | Present | 136 (23.9) | 174 (19.9) | 310 |  | 1.145 | 0.990-1.325 |  |
| Neoplasia | Absent | 537 (94.2) | 836 (95.8) | 1,373 | 0.210 | Reference | - |  |
|  | Present | 33 (5.8) | 37 (4.2) | 70 |  | 1.205 | 0.933-1.558 |  |
| Thyreopathy | Absent | 535 (93.9) | 838 (96.0) | 1,373 | 0.079 | Reference | - |  |
|  | Present | 35 (6.1) | 35 (4,0) | 70 |  | 1.283 | 1.006-1.637 |  |
| Kidney disease | Absent | 534 (93.7) | 849 (97.3) | 1,383 | 0.001 | Reference | - |  |
|  | Present | 36 (6,3) | 24 (2.7) | 60 |  | 1.554 | 1.251-1.931 |  |
| Hepatopathy | Absent | 561 (98.4) | 864 (99.0) | 1,425 | 0.467 | Reference | - |  |
|  | Present | 9 (1.6) | 9 (1.0) | 18 |  | 1.270 | 0.797-2.025 |  |
| Neurologic Sequel | Absent | 511 (89.6) | 761 (87.2) | 1,272 | 0.158 | Reference | - |  |
|  | Present | 59 (10.4) | 112 (12.8) | 171 |  | 0.859 | 0.691-1.067 |  |
| Immunosuppression | Absent | 557 (97.7) | 861 (98.6) | 1,418 | 0.219 | Reference | - |  |
|  | Present | 13 (2.3) | 12 (1.4) | 25 |  | 1.324 | 0.903-1.940 |  |
| Gastrointestinal disorder | Absent | 565 (99.1) | 862 (98.7) | 1,427 | 0.612 | Reference | - |  |
|  | Present | 5 (0.9) | 11 (1.3) | 16 |  | 0.789 | 0.381-1.697 |  |
| Other clinical condition^*^ | Absent | 553 (97.0) | 845 (96.8) | 1,398 | 0.878 | Reference | - |  |
|  | Present | 17 (3.0) | 28 (3.2) | 45 |  | 0.955 | 0.653-1.397 |  |
| Patient’s age was associated with the presence of drug use [*p*<0.001; (Yes) 35.12±11.42, 34 (26.5 to 40); (No) 57.47±17.24; 60 (47 to 70)] and alcoholism [*p*=0.013; (Yes) 55.43±13.37, 57 (47 to 64); (No) 56.95±18.20; 60 (45 to 70)], and individuals who presented drug use and alcoholism were younger. N, number of individuals; %, percentage; RR, relative risk; 95%CI, 95% confidence interval. ^*^, Supplementary Table 1. | | | | | | | | |

| **Table 4.** Association between patient’s origin, diagnosis indicating intubation, presence of pneumonia associated with invasive mechanical ventilation, need for tracheostomy, and intubation markers of patients admitted to the intensive care unit on invasive mechanical ventilation as a death risk factor. | | | | | | | |
| --- | --- | --- | --- | --- | --- | --- | --- |
| **Patient’s characteristics** | **Group** | **Death – N (%)** | **Discharge – N (%)** | **Total – N** | ***p*** | **RR** | **95%CI** |
| Origin | Surgery | 320 (56.1) | 603 (69.1) | 923 | 0.001 | Reference | - |
|  | Clinic | 250 (43.9) | 270 (30.9) | 520 |  | 1.387 | 1.223-1.573 |
| Diagnosis |  |  |  |  |  |  |  |
| Traumatic brain injury | Absent | 510 (89.5) | 736 (84.3) | 1,246 | 0.006 | Reference | - |
|  | Present | 60 (10.5) | 137 (15.7) | 197 |  | 0.744 | 0.596-0.928 |
| Polytrauma | Absent | 512 (89.8) | 721 (82.6) | 1,233 | 0.001 | Reference | - |
|  | Present | 58 (10.2) | 152 (717.4) | 210 |  | 0.665 | 0.290-0.836 |
| Sepsis | Absent | 383 (67.2) | 685 (78.5) | 1,068 | 0.001 | Reference | - |
|  | Present | 187 (32.8) | 188 (21.5) | 375 |  | 1.391 | 1.222-1.583 |
| Elective Surgery | Absent | 379 (66.5) | 448 (51.3) | 827 | 0.001 | Reference | - |
|  | Present | 191 (33.5) | 425 (48.7) | 616 |  | 0.677 | 0.589-0.778 |
| Acute Myocardial Infarction | Absent | 527 (92.5) | 827 (94.7) | 1,354 | 0.093 | Reference | - |
|  | Present | 43 (7.5) | 46 (5.3) | 89 |  | 1.241 | 0.991-1.555 |
| Stroke | Absent | 502 (88.1) | 820 (93.9) | 1,322 | 0.001 | Reference | - |
|  | Present | 68 (11.9) | 53 (6.1) | 121 |  | 1.480 | 1.246-1.757 |
| Subarachnoid hemorrhage | Absent | 520 (91.2) | 819 (93.8) | 1,339 | 0.076 | Reference | - |
|  | Present | 50 (8.8) | 54 (6.2) | 104 |  | 1.238 | 1.003-1.528 |
| Neoplasia | Absent | 562 (98.6) | 858 (98.3) | 1,420 | 0.675 | Reference | - |
|  | Present | 8 (1.4) | 15 (1.7) | 23 |  | 0.879 | 0.500-1.544 |
| Neurologic and psychiatric disease | Absent | 550 (96.5) | 824 (94.4) | 1,374 | 0.077 | Reference | - |
|  | Present | 20 (3.5) | 49 (5.6) | 69 |  | 0.724 | 0.498-1.053 |
| Cardiopathy | Absent | 471 (82.6) | 750 (85.9) | 1,221 | 0.101 | Reference | - |
|  | Present | 99 (17.4) | 123 (14.1) | 222 |  | 1.156 | 0.982-1.730 |
| Kidney disorder | Absent | 552 (96.8) | 860 (98.5) | 1,412 | 0.040 | Reference | - |
|  | Present | 18 (3.2) | 13 (1.5) | 31 |  | 1.485 | 1.094-2.017 |
| Other diagnosis^**^ | Absent | 545 (95.6) | 849 (97.3) | 1,394 | 0.103 | Reference | - |
|  | Present | 25 (4.4) | 24 (2.7) | 49 |  | 1.305 | 0.984-1.730 |
| Pneumonia associated with invasive mechanical ventilation | Absent | 412 (72.3) | 621 (71.1) | 1,033 | 0.676 | Reference | - |
|  | Present | 158 (27.7) | 252 (28.9) | 410 |  | 0.966 | 0.837-1.115 |
| Tracheostomy | Absent | 478 (83.9) | 633 (72.5) | 1,111 | 0.001 | Reference | - |
|  | Present | 92 (16.1) | 240 (27.53) | 332 |  | 0.644 | 0.535-0.776 |
| Oxygen blood pressure (PaO_2_) | Hypoxia | 85 (14.9) | 57 (6.5) | 142 | <0.001 | 1.365 | 1.126-1.655 |
|  | Normal | 114 (20.0) | 146 (16.7) | 260 |  | Reference | - |
|  | Hyperoxia | 371 (65.1) | 670 (76.7) | 1,041 |  | 0.813 | 0.693-0.954 |
| Positive end-expiratory pressure | ≤8 cmH_2_O | 486 (85.3) | 818 (93.7) | 1,304 | <0.001 | Reference | - |
|  | >8 cmH_2_O | 84 (14.7) | 55 (6.3) | 139 |  | 1.621 | 1.393-1.887 |
| The patients’ age was associated with the presence of traumatic brain injury [*p*<0.001; (Yes) 40.87±17.06, 38 (25 to 36); (No) 59.22±16.28; 61 (50 to 71)] and polytrauma [*p*<0.01; (Yes) 38.73±16.62, 34 (24.75 to 51); (No) 59.78±15.78; 61 (51 to 71)], and individuals who presented traumatic brain injury and polytrauma were younger. N, number of individuals; %, percentage; RR, relative risk; 95%CI, 95% confidence interval. ^**^, Supplementary Table 2. | | | | | | | |

**Figure 1.** Flowchart of medical record analysis and inclusion of intubated patients in the intensive care unit on invasive mechanical ventilation of a University Hospital in São Paulo State, Brazil. N, number of patients.

**Figure 2.** Pearson’s correlation between markers with numeric distribution [positive end-expiratory pressure (PEEP) at admission, arterial oxygen pressure (PaO_2_) at admission, time receiving invasive mechanical ventilation (IMV), hospitalization (hospital stay) time, time up to the diagnosis of ventilation-associated pneumonia (VAP), body mass index (BMI), and age] included in the study. In the Spearman correlations, we considered the following cut-off points: (i) ±0.90–1.00, very strong positive–negative correlation index; (ii) ±0.70–0.89, strong positive–negative correlation index; (iii) ±0.40–0.69, moderate positive–negative correlation index; (iv) ±0.10–0.39 weak positive–negative correlation index; and (v) 0.00–0.09, insignificant (negligible) positive–negative correlation index. We adopted an alpha error of 0.05.
